# Genome Analysis of the Probiotic Strain Enterococcus faecium Ef79OSAU

**DOI:** 10.1128/MRA.00691-21

**Published:** 2021-10-21

**Authors:** M. V. Sycheva, E. V. Popova, Y. A. Khlopko, T. M. Pashkova, O. L. Kartashova, V. Y. Kataev, V. V. Gerasimenko, A. V. Vasilchenko

**Affiliations:** a Orenburg State Agrarian University, Orenburg, Russia; b Institute of Cellular and Intracellular Symbiosis, Ural Branch of the Russian Academy of Sciences, Orenburg, Russia; c Laboratory of Antimicrobial Resistance, Institute of Environmental and Agricultural Biology (X-BIO), Tyumen State University, Tyumen, Russia; d All-Russian Institute of Plant Protection, St. Petersburg-Pushkin, Russia; Loyola University Chicago

## Abstract

We report here the draft genome sequence of Enterococcus faecium strain Ef79OSAU, which was isolated from swine feces. The characteristics of strain Ef79OSAU reveal the absence of pathogenicity factors, a wide range of antimicrobial activity *in vitro*, and antilisteriosis activity *in vivo*. Analysis of the E. faecium Ef79OSAU genome revealed a cluster of genes encoding enterocin A without genetic determinants of pathogenicity.

## ANNOUNCEMENT

Enterococcus faecium strain Ef79OSAU was isolated from the feces of healthy swine (Orenburg, Russia) using bacteriological approaches (1). The feces were dispersed in sterile isotonic (0.85% NaCl) solution, then added at 10-fold dilutions to the surface of differential diagnostic bile-esculin medium with sodium azide (HiMedia, India) and incubated at 37°C for 24 h. The microorganisms were identified as a species using multiplex PCR to ascertain the presence of species-specific genes encoding superoxide dismutase synthesis (2).

A colony from a pure culture plate of strain Ef79OSAU was inoculated into 5 ml Luria-Bertani medium and incubated at 37°C for 12 h. Genomic DNA was extracted using a Quick-DNA fungal/bacterial kit (Zymo Research, USA). The quality of the extracted DNA was assessed using the *A*_260/280_ ratio with the NanoDrop 8000 spectrophotometer (Thermo Fisher Scientific, USA), and electrophoresis was performed in 1% agarose gel. The DNA concentration was quantified using the Qubit 4 fluorometer and a double-stranded DNA (dsDNA) high-sensitivity assay kit (Life Technologies, USA).

A DNA library was prepared for whole-genome sequencing using a NEBNext Ultra II FS DNA library prep kit for Illumina (New England Biolabs, USA).

The library was validated using capillary electrophoresis on a QIAxcel Advanced system (Qiagen, Germany) using the QIAxcel DNA high resolution kit and normalized using quantitative PCR (qPCR) on the CFX Connect real-time PCR system (Bio-Rad, USA).

As a result of sequencing, 7,099,520 paired-end reads were obtained. Paired-end sequencing (2 × 300 bp) was carried out on a MiSeq platform (Illumina, USA) using a reagent kit v3 (Illumina) at the “Persistence of Microorganisms” Center of Shared Scientific Equipment of the Institute for Cellular and Intracellular Symbiosis, UB RAS.

The sequencing data were analyzed using FastQC v0.11.17 software. Based on this analysis, the original reads were trimmed using the Trimmomatic v0.36 program (3) with the parameters LEADING: 30, TRAILING: 30, SLIDINGWINDOW: 15:30, HEADCROP: 12, ILLUMINACLIP: adapters.fa:2:30: and MINLEN: 30. The remaining reads were reanalyzed using FastQC v0.11.17. The genome was assembled *de novo* using the genome assembler SPAdes v3.14.0 (4) with the careful option.

The assembly yielded 72 contigs, available at the National Center for Biotechnological Information (NCBI) GenBank, with a total length of 2,644,772 bp, an *N*_50_ value of 266,117 bp, and an *L*_50_ value of 5. It has a G+C content of 38.0% and an average coverage of 577.0×. The genome sequence was annotated using the NCBI Prokaryotic Genome Annotation Pipeline (PGAP) v5.2 (5) with default settings. The annotation identified 2,607 coding sequences, including 2,433 proteins, 74 pseudogenes, 34 rRNA genes (4 5S, 26 16S, and 4 23S), 62 tRNA genes, and 4 noncoding RNA genes (ncRNAs).

Genome annotation was also performed using the antiSMASH v6 (6) and RAST (7) servers with default settings. An enterocin A biosynthetic gene cluster (BGC) was found (location, nucleotides 84799 to 107917) that has full homology with the BGC of Enterococcus faecium DO (NCBI GenBank accession number CP003583.1). According to the RAST annotation results, the genome of E. faecium Ef79OSAU has no genes encoding the synthesis of known pathogenicity factors.

Thus, the resulting draft genome sequence and the analysis of its functional annotation determine the applied interest in the studied isolate. In particular, E. faecium Ef79OSAU is a promising candidate for inclusion in probiotic biologics.

### Data availability.

This whole-genome shotgun project has been deposited in GenBank under the accession number JAHQCZ000000000. The version described in this paper is JAHQCZ010000000. The raw sequence data are publicly available under SRA accession number SRR14879700.
